# Macaque structural connectivity revisited: CoCoMac 2.0

**DOI:** 10.1186/1471-2202-12-S1-P72

**Published:** 2011-07-18

**Authors:** Rembrandt Bakker, Tobias C Potjans, Thomas Wachtler, Markus Diesmann

**Affiliations:** 1 Donders Inst. for Brain, Cognition and Behaviour, Radboud University Nijmegen, Netherlands; 2 Institute of Neuroscience and Medicine (INM-6), Research Center Jülich, Germany; 3 Department Biology II, Ludwig-Maximilians-Universität München, 82152 Planegg-Martinsried, Germany; 4 RIKEN Computational Science Research Program, Wako-shi, Saitama, Japan; 5 RIKEN Brain Science Institute, Wako-shi, Saitama, Japan

## 

CoCoMac (cocomac.org) is a large data base on structural connectivity in the Macaque brain, based on over 450 published axonal tracing studies [1]. This huge curation effort took place under the guidance of Rolf Kötter, and provided data to numerous brain network analysis and modeling studies. While working on a major new release of the database, Rolf Kötter sadly passed away in June 2010. We are committed to foster the further development of CoCoMac and are gradually releasing the newly developed web interface at the CoCoMac 2.0 server hosted at the German INCF Node (cocomac.g-node.org).

Macaque structural connectivity data is relevant for uncovering the large-scale human connectome and increasingly plays a role in constraining neuronal network models that link the connectivity structure to activity dynamics and network function. With recent advances in computer hardware and simulation software [2], brain-scale simulations with millions of spiking neurons become feasible, accounting simultaneously for the macroscopic and the microscopic structure of cortical networks. These models promote the integration of local micro circuitry and long-range connectivity data on the level of the cell-type specificity of connections. Such level of detail cannot be provided by diffusion MRI-based techniques: for layer-specificity, intracortical resolution and directionality, one has to fall back to axonal tracing experiments. Combining these into a complete picture for the entire brain is an enormous challenge, as it involves experiments that have been measured in thousands of individual brains over the course of a century.

Since the spatial coordinates of tracing injections are undocumented in most publications, CoCoMac has had no other choice than to describe connectivity completely in terms of named brain regions: ‘region A has axonal projections to region B’. A nomenclature mapping service known as ORT [3] translates named brain regions in older brain atlases to newer ones and vice versa. Producing correct nomenclature mappings is of crucial importance. Incorrect or imprecise use of nomenclature in the literature leads to conflicting (chains of) mapping statements, and to errors in the resulting connectivity. CoCoMac 2.0 detects for each mapping statement whether conflicting versions exist, and uses Bayesian reasoning to eliminate the inconsistent literature statements causing the conflicts.

For data exchange with MRI-based techniques, CoCoMac needs to attach spatial coordinates to its connectivity data. This is achieved by integrating CoCoMac with the INCF Scalable Brain Atlas (SBA, scalablebrainatlas.incf.org/cocomac) [4]. This web-based tool interactively displays structural connectivity in a spatial reference framework, and supports a number of commonly used brain atlases. The SBA provides a point-and-click interface to the low level text-based services at the CoCoMac server.

## References

[B1] Kötter R Online retrieval, processing, and visualization of primate connectivity data from the CoCoMac database Neuroinformatics 2004 2 127 144 10.1385/NI:2:2:127 15319511

[B2] Potjans TC Kunkel S Morrison A Plesser HE Diesmann M Simulating neuronal networks at the brain scale on BlueGene/P supercomputers with NEST Soc. for Neurosci. Meeting, San Diego, CA 2010 208 29

[B3] Stephan KE Zilles K Kötter R Coordinate-independent mapping of structural and functional data by objective relational transformation (ORT) Philos Trans R Soc Lond B Biol Sci 2000 355 1393 37 54 10703043 10.1098/rstb.2000.0548 PMC1692724

[B4] Bakker R Bezgin G Kötter R Brain connectivity at your fingertips: CoCoMac interfaced with the Scalable Brain Atlas Soc. for Neurosci. Meeting, San Diego, CA 2010 230 08

